# Steroid sulfatase is a potential modifier of cognition in attention deficit hyperactivity disorder

**DOI:** 10.1111/j.1601-183X.2010.00672.x

**Published:** 2011-04

**Authors:** E Stergiakouli, K Langley, H Williams, J Walters, N M Williams, S Suren, I Giegling, L S Wilkinson, M J Owen, M C O'Donovan, D Rujescu, A Thapar, W Davies

**Affiliations:** † MRC Centre for Neuropsychiatric Genetics and Genomics and Department of Psychological Medicine and Neurology, School of Medicine, Cardiff UniversityCardiff; ‡ School of Psychology, Cardiff UniversityCardiff; § Human Developmental Biology Resource, University College London Institute of Child HealthLondon, UK; ¶ Department of Psychiatry, Section of Molecular and Clinical Neurobiology, Ludwig Maximilians UniversityMunich, Germany; ** Behavioural Genetics Group, Schools of Medicine and Psychology, Cardiff UniversityCardiff, UK

**Keywords:** Association, attention, brain, cognition, dehydroepiandrosterone sulfate, neurosteroid, picture completion, testosterone, Wechsler Intelligence scales

## Abstract

Deletions encompassing the X-linked *STS* gene (encoding steroid sulfatase) have been observed in subjects with neurodevelopmental disorders, including attention deficit hyperactivity disorder (ADHD). Recently, two single nucleotide polymorphisms (SNPs) within *STS* (rs12861247 and rs17268988) have been reported to be associated with ADHD risk and inattentive symptoms in ADHD, respectively. Using a UK sample of ADHD subjects (aged 5–18 years), we tested the hypothesis that rs12861247 is associated with ADHD risk using a case–control approach (comparing 327 ADHD cases with 358 male controls from the Wellcome Trust Case Control Consortium). Using a subset of males from the ADHD sample, we also examined whether variation within *STS* is associated with symptomatology/cognitive function in ADHD. We then tested whether SNPs associated with cognitive function in ADHD were also associated with cognitive function in healthy male subjects using a German sample (*n* = 143, aged 18–30 years), and whether *STS* was expressed in brain regions pertinent to ADHD pathology during development. We did not replicate the previously identified association with rs12861247. However, in ADHD males, variation at rs17268988 was associated with inattentive symptoms, while variation within *STS* was significantly associated with performance on three cognitive measures. Three SNPs associated with cognitive function in ADHD males were not associated with cognitive function in healthy males. *STS* was highly expressed in the developing cerebellar neuroepithelium, basal ganglia, thalamus, pituitary gland, hypothalamus and choroid plexus. These data suggest that genetic variants affecting STS expression and/or activity could influence the function of brain regions perturbed in ADHD.

Cytogenetic deletions on the short arm of the X chromosome encompassing the steroid sulfatase (*STS*) gene have been noted in cases of neurodevelopmental disorders associated with abnormal cognition, including attention deficit hyperactivity disorder (ADHD) (Doherty *et al.* 2003; Lonardo *et al.* 2007; Tobias *et al.* 2001), schizophrenia (Milunsky *et al.* 1999) and autism (Shinawi *et al.* 2009; Thomas *et al.* 1999; Vorstman *et al.* 2006). These findings suggest the possibility that a lack of the brain-expressed protein encoded by *STS*, the enzyme steroid sulfatase, might influence vulnerability to, or the presentation of, these particular disorders. The function of STS is to cleave sulfate groups from a variety of steroid hormones [e.g. dehydroepiandrosterone sulfate (DHEAS)], thereby modulating their neural function and activity (Reed *et al.* 2005). Dehydroepiandrosterone sulfate and its non-sulfated form DHEA are metabolized to a number of biologically significant molecules including testosterone (Reed *et al.* 2005), and influence key neurodevelopmental processes (Herbert 1998). Abnormal DHEA(S) levels have been implicated in the pathogenesis of a number of neuropsychiatric disorders, including schizophrenia (Maninger *et al.* 2009).

The link between STS dysfunction and ADHD (a disorder associated with attention deficits, impulsivity, hyperactivity, cognitive abnormalities and aggression in some subjects) has been strengthened by four observations: first, subjects with deletions of *STS*, or with inactivating mutations within the gene (and thus presenting with the dermatological condition X-linked ichthyosis, XLI), are at significantly increased risk of developing ADHD (Kent *et al.* 2008). Second, two single nucleotide polymorphisms (SNPs) within *STS* (rs2270112 and rs12861247) have been reported to be associated with ADHD, although only the latter survived correction for multiple testing; in the same study SNP rs17268988 was associated with inattentive symptoms (Brookes *et al.* 2008). Third, systemic levels of DHEA appear to correlate with ADHD symptomatology, while methylphenidate treatment may exert its therapeutic effect in ADHD through increasing DHEA levels (Golubchik *et al.* 2007). Finally, deletion of the *STS* gene in mice (or inhibition of the associated enzyme) is associated with attention deficits, with alterations in impulsivity, and with elevated aggression (Davies *et al.* 2009; Mortaud *et al.* 2010; Nicolas *et al.* 2001).

The aims of the present study were fourfold: (1) to try and replicate the most robust association finding previously identified with rs12861247 using a case–control protocol, (2) to investigate how variation across the *STS* gene might influence symptomatology and general cognitive function in ADHD subjects; with regard to effects on symptomatology, we focussed on possible association with inattentive, impulsive and aggressive symptoms given the previous findings in mouse and man, (3) to ascertain whether variants associated with symptomatology and/or cognitive function in an ADHD sample were also associated with effects on cognition in healthy volunteers and (4) to identify the pattern of expression of *STS* in the developing human brain with a view to understanding how STS activity might influence normal neurodevelopment, and thereby to understand whether there are plausible biological underpinnings to any observed genetic associations with cognitive endophenotypes.

## Materials and methods

### Case–control study: ADHD and control samples

A sample of 393 British Caucasian subjects in total (352 males, 41 females) between the ages of 5 and 18 years who had recently been diagnosed with ADHD was recruited from Child and Adolescent Psychiatrists and Paediatricians in the Greater Manchester, South Wales and Avon areas of the United Kingdom. The presence of symptoms and research diagnoses of Diagnostic and Statistical Manual of Mental Disorders, Version IV (DSM-IV ) ADHD and comorbid conditions were obtained from parent reports using the Child and Adolescent Psychiatric Assessment (CAPA) (Angold & Costello 2000), with pervasiveness of ADHD assessed using the Child ADHD Teacher Telephone Interview (ChATTI) (Holmes *et al.* 2004). All assessments were conducted by psychology graduates trained to high levels of inter-rater reliability. Individuals were excluded from the study if they had a full-scale IQ < 70, Tourette's syndrome, any neurological condition (including epilepsy) or a pervasive developmental disorder. To further assess this last criterion, participants were also assessed using the Autism Screening Questionnaire (Berument *et al.* 1999), and those scoring >14 were excluded from the study. All individuals met DSM-IV criteria for ADHD. The overall composition of our sample was 6% inattentive subtype, 81% combined subtype and 13% hyperactive-impulsive subtype, rates consistent with a previous study examining association in *STS* in UK and Irish ADHD samples (Brookes *et al.* 2008). Subjects with ADHD were genotyped at rs12861247 for the case–control analysis.

The control sample genotyped at rs12861247 for the case–control study (hereafter referred to as the control group) consisted of 360 males from the Wellcome Trust Case Control Consortium 1958 Birth Cohort sample, used in previous association studies in Cardiff (Wellcome Trust Case Control Consortium 2007). These control subjects, like the ADHD sample, comprise British Caucasians, and are thought to faithfully represent the genome of this population, with little evidence for population stratification (Wellcome Trust Case Control Consortium 2007). Sufficient control subjects were genotyped such that the combined sample size was calculated *a priori* to give a power of >80% to detect an odds ratio of >1.52, and a power of 100% to detect an odds ratio of >2.00, assuming a disorder prevalence of 6% (Thapar *et al.* 2005) (Brookes *et al.* 2008, association of rs12861247 with ADHD gave rise to an odds ratio of 2.05, with a lower 95% confidence interval of 1.88).

### Symptomatology and cognitive analysis in ADHD subjects

Of the overall ADHD sample described above, a total of 266 male subjects were used to assay the effects of *STS* variation on symptomatology and cognition. Importantly, given the possible long- and short-term effects of medication on behavior and cognition, these subjects had been on medication for <1 year, and had been medication-free for at least 24 h prior to assessment. The mean age of this subset of subjects was 111.5 ± 1.5 months.

Using the CAPA, the number of parent-reported DSM-IV inattentive symptoms (maximum nine) and impulsive symptoms (maximum four) was ascertained (hyperactivity symptoms were also assessed, but were not analyzed here as there was no prior evidence for STS effects on this measure). The presence of DSM-IV aggressive conduct disorder symptoms, again ascertained using the CAPA, was also investigated. This subgroup of conduct disorder symptoms consists of seven measures: (1) often bullies, threatens or intimidates others, (2) often initiates physical fights, (3) has used a weapon that can cause serious physical harm to others, (4) has been physically cruel to people, (5) has been physically cruel to animals, (6) has stolen while confronting a victim and (7) has forced someone into sexual activity. The final measure was not assessed in this sample, resulting in a maximum of six possible aggressive symptoms being present.

Cognitive performance in this sample was tested using the Wechsler Intelligence Scales for Children Version III (WISC-III; Wechsler 1991). This assessment of cognitive ability consists of 13 subtests, 10 of which are used to calculate a standardized full-scale IQ score. Based on previous studies discriminating individuals with ADHD from control subjects using Wechsler scales (Antshel *et al.* 2007; Ek *et al.* 2007; Filippatou & Livaniou 2005; Mayes & Calhoun 2004; Pineda *et al.* 1999), we investigated performance on six measures by genotype: information, coding, block design, freedom from distractibility (derived from scores on the arithmetic and digit span subtests), picture completion and comprehension. We also examined performance on two general measures of cognition (performance IQ and verbal IQ) by genotype. Parents provided written informed consent, and children informed assent, prior to the assessments. The studies involving ADHD subjects were approved by North West and Wales Multicentre Research Ethics Committees and local National Health Service Research and Development offices.

### Healthy volunteer samples and assessments

For this study, all available male participants aged 18–30 years (total *n* = 143, hereafter referred to as the healthy volunteer group) were drawn from a sample of 2337 healthy volunteers of German descent who were randomly selected from the general population of Munich, Germany, and contacted by mail. To exclude subjects with central neurological diseases and psychotic disorders or subjects who had first-degree relatives with psychotic disorders, several screenings were conducted before the volunteers were enrolled in our study. First, subjects who responded were initially screened by telephone for the absence of neuropsychiatric disorders. Second, detailed medical and psychiatric histories were assessed for both themselves and their first-degree relatives by using a semistructured interview. Third, if no exclusion criteria were fulfilled, the subjects were invited to a comprehensive interview including the Structured Clinical for DSM-IV Axis I Disorders – Patient Edition (First *et al.* 1995) and the Structured Clinical Interview for DSM-IV Axis II Personality Disorders (First *et al.* 1990) to validate the absence of any lifetime psychotic disorder. In addition, the Family History Assessment Module (Rice *et al.* 1995) was conducted to exclude psychotic disorders among first-degree relatives. A neurological examination was also conducted to exclude subjects with current central nervous system impairment. Subjects were administered the German version of the Wechsler Adult Intelligence Scale-Revised (WAIS-R; Tewes 1991, the adult equivalent of the WISC-III) using all 11 subtests (vocabulary, comprehension, information, digit span, arithmetic, similarities, block design, picture completion, picture arrangement, object assembly and digit symbol coding). The recruitment, genotyping and behavioral testing of the healthy volunteer participants were approved by the Institutional Review Board of the Ludwig-Maximilians-University of Munich, Germany.

### Genotyping in ADHD and control samples

Genomic DNA was extracted from saliva or whole blood samples according to routine procedures. Single-nucleotide polymorphism rs12861247 was genotyped in the ADHD sample and in the control group using TaqMan SNP genotyping assay C_321340_10 according to the manufacturer's instructions (Applied Biosystems, Warrington, UK).

Subjects for which symptomatology and cognitive data are presented were genotyped for eight Tag-SNPs covering the promoter and coding regions of the *STS* gene (Table 1). Single-nucleotide polymorphisms were chosen using Haploview Tagger (Barrett *et al.* 2005) at *r*^2^ = 0.8 in order to achieve widespread coverage of the gene locus (tagging ∼68% of the *STS* gene). Six of these SNPs (with the exceptions of rs12861247 and rs5978405) were genotyped in 233 ADHD males using a Sequenom MassARRAY system and Sequenom iPLEX GOLD chemistry according to the manufacturer's instructions. Assays were designed using Assay Design software version 3.1 (Sequenom Inc., San Diego, CA, USA) and genotyping was performed using Typer software version 4.0 (Sequenom Inc.). All thermocycling reactions were performed using an MJ Thermocycler. Specific details of reaction conditions and primers are available on request. Quality control assessment of genotyping assays after optimization involved analysis in the CEU individuals genotyped as part of the HapMap project prior to genotyping in the association sample (http://www.hapmap.org). Only assays with 100% concordance with HapMap genotypes were analyzed further. All association sample plates contained cases, controls, blanks and CEU samples. Genotypes were called in duplicate blind to sample identity and blind to the other rater. Single-nucleotide polymorphism rs12861247 was genotyped as described above. rs5978405 was genotyped using TaqMan SNP genotyping assay C_30005846_10 according to the manufacturer's instructions (Applied Biosystems). Linkage disequilibrium analysis was performed using Haploview (Barrett *et al.* 2005).

**Table 1:** Allelic frequency at SNPs across the *STS* promoter and coding regions

SNP assayed	Location of SNP within gene	Minor allele frequency from HapMap CEU sample (both sexes)/males only	Minor allele frequency from Brookes *et al.* (2008) Dublin ADHD sample	Minor allele frequency from Brookes *et al.* (2008) St Andrews ADHD sample	Minor allele frequency from present ADHD sample	Minor allele frequency from present healthy volunteer sample
rs5934671	Putative promoter	0.33/0.27	N/A	N/A	0.34	N/A
rs2270112	Intron 1	0.26/0.33	0.27	0.26	0.31	N/A
rs12861247	Intron 2	0.20/0.20	0.10	0.09	0.10	N/A
rs5978405	Intron 6	0.44/0.40	N/A	N/A	0.41	0.37
rs17268974	Intron 7	0.24/0.21	N/A	N/A	0.24	N/A
rs4403552	Intron 7	0.30/0.30	0.25	0.24	0.21	N/A
rs17268988	Intron 9	0.20/0.27	0.21	0.21	0.24	0.35
rs5933863	3′-UTR	0.22/0.20	0.16	0.16	0.17	0.11

3′-UTR, 3′-untranslated region.

HapMap minor allele frequencies are reported for combined male and female samples (*n* = 60) and for male founder samples alone (*n* = 30). Each of the eight SNPs assayed in our ADHD sample was genotyped in ≥179 males, and each of the three SNPs assayed in the healthy volunteer group was genotyped in ≥134 males.

### Genotyping in healthy volunteer samples

Three SNPs, rs5978405, rs5933863 and rs17268988, which showed evidence for association (replicated in the case of rs17268988) with symptomatology/cognitive measures in the ADHD sample, were successfully genotyped in healthy volunteer subjects using the Sequenom MassARRAY system; rs12861247, which also showed strong evidence for association, could not be typed using this panel. Genomic DNA was extracted from whole blood samples according to routine procedures. One nanogram of DNA was assayed using the iPLEX assay on the MassARRAY MALDI-TOF mass spectrometer (SEQUENOM, Hamburg, Germany). DNA concentration was adjusted using the PicoGreen quantitation reagent (Invitrogen, Karlsruhe, Germany). Quality control assessment of genotyping assays involved analysis in the CEU individuals genotyped as part of the HapMap project (http://www.hapmap.org). Only assays with 100% concordance with HapMap genotypes were analyzed further.

### Statistical analysis

The case–control data were analyzed using plink 1.06, which takes into account the location of the SNPs on the X chromosome (Purcell *et al.* 2007). Behavioral and cognitive data are presented as mean values ± standard error of the mean, and were analyzed using spss 16. As symptomatology data were not normally distributed, they were analyzed by Mann–Whitney *U* test or Kruskal–Wallis test to test for allelic association. Wechsler scale subtest scores were tested for statistical normality by Shapiro–Wilk's test, transformed if appropriate, and then subjected to two-tailed *t*-tests to compare allelic effects. Scores which were not normally distributed and which could not be transformed to approximate a normal distribution were compared using the Mann–Whitney *U* test. Nominal *P* values are reported. *P* values of <0.05 following extremely conservative Bonferroni correction for multiple testing (taking into account the number of SNPs genotyped and the number of phenotypes assayed) were regarded as significant (Goodarzi *et al.* 2007), i.e. *P* values <0.00078 prior to correction were regarded as significant in the ADHD sample analysis, while *P* values <0.0024 prior to correction were regarded as significant in the healthy volunteer sample.

### In situ hybridization

A polymerase chain reaction (PCR) product corresponding to NM_000351.3 (bp 2570–3249) was subcloned into pGEM-T Easy vector (Promega, Southampton, UK). The vector was linearized using *Sal*I or *Nco*I restriction enzymes (Promega), and transcribed using T7 or Sp6 polymerases to generate antisense or sense ribonucleotide probes, respectively. Digoxigenin-uridine triphosphate was incorporated into riboprobes during *in vitro* transcription using DIG RNA labeling mix (Roche Diagnostics, Burgess Hill, UK) according to the manufacturer's instructions. Labeled probes were then hybridized to human embryonic or fetal tissue obtained from the Human Developmental Biology Resource (University College London Institute of Child Health, London, UK) according to standard protocols (Wilkinson 1992).

## Results

### Case–control analysis

For our case–control study, a total of 327 ADHD cases (290 males, 37 females) and 358 male control subjects were successfully genotyped for rs12861247. The frequency of the minor allele (A) at this SNP did not differ significantly between the two groups: 0.107 in cases (0.097 in males and 0.149 in females) and 0.101 in control subjects, *P* = 0.762, odds ratio = 1.08, 95% confidence interval 0.667–1.737. When the analysis was limited to males only, again there was no significant difference between cases and controls (*P* = 0.888, odds ratio = 0.96, 95% confidence interval 0.570–1.614). Data from the females genotyped indicated that there was no significant deviation from Hardy–Weinberg equilibrium at this SNP (27 GG homozygotes, 9 GA heterozygotes, 1 AA homozygote, two-tailed Fisher's exact test, *P* = 1.0).

### Genotyping and allelic frequency in the ADHD sample

**Figure 1:**
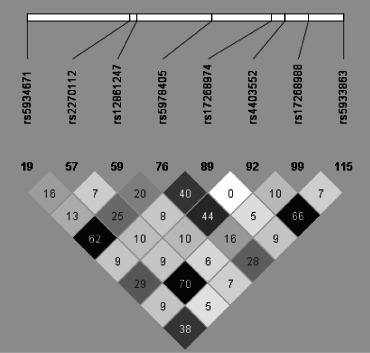
The *r*^2^ linkage disequilibrium between typed markers across the *STS* locus in our ADHD sample The Tag SNP markers analysed were not in high linkage disequilibrium with one another.

Our genotyping success rate across all eight SNPs analyzed in the ADHD sample for which behavioral/cognitive data are presented was 90.5 ± 2.6%. The minor allele frequencies we observed were comparable with those seen previously in British and Irish ADHD samples (Brookes *et al.* 2008), and with those reported in residents of Utah with White European ancestry (the CEU population) in the HapMap database (Table 1). The Tag SNP markers that we analyzed were not in high-linkage disequilibrium with one another (Fig. 1).

### Genotyping and allelic frequency in the healthy volunteer sample

Our genotyping success rate across the three SNPs analyzed in the healthy volunteer sample was 96.3 ± 1.3%. Minor allele frequencies within the healthy volunteer sample are shown in Table 1.

### Association between STS variants and symptomatology in male ADHD subjects

**Figure 2:**
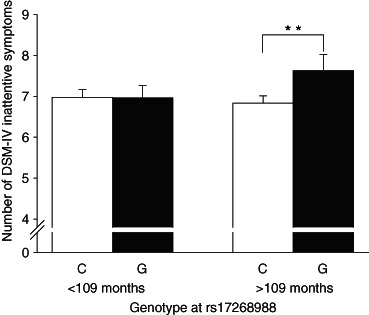
Relationship between genotype at rs17268988, age and DSM-IV inattentive symptoms in ADHD males In ADHD males aged <109 months, genetic variation at rs17268988 appeared to have no effect on the number of DSM-IV inattentive symptoms (C: *n* = 79, G: *n* = 24). However, in subjects ≥109 months, the presence of a G allele at this SNP (*n* = 27) was associated with a significantly greater number of inattentive symptoms than the presence of a C allele (*n* = 84) (**P≤ 0.005).

As the Y-linked homolog of *STS* is a non-expressed pseudogene (Yen *et al.* 1988), males will be hemizygous for *STS* alleles; hence, we initially looked for association in males, in that genotype–phenotype correlations should be easier to interpret in this sex. We found nominally significant evidence for allelic variation at one SNP (rs17268988) being associated with the number of DSM-IV inattentive symptoms, with males possessing a G allele (*n* = 51) showing more inattentive symptoms than those possessing the more common C allele (*n* = 163) (*U* = 4906, nominal *P* value = 0.026; Table 2). Importantly, the ages of the G- and C-allele groups were equivalent (Table S1). Although this result did not remain significant upon correcting for multiple comparisons, as it replicated a previous observation (Brookes *et al.* 2008), we decided to follow it up further. Specifically, given that ADHD is a developmental disorder characterized by symptom reduction with age (Langley *et al.* 2009), we tested whether the effect of the SNP on inattentive symptoms was age dependent, by subdividing our male sample in two around the median age of 109 months: subjects <109 months of age (C: *n* = 79, G: *n* = 24) and subjects ≥109 months of age (C: *n* = 84, G: *n* = 27); this resulted in two evenly sized groups (and hence maximal power to detect an age-dependent effect). We found no effect of allelic variation on inattentive symptoms in the younger subgroup (*U* = 915, *P* = 0.80), but a significant effect in the older subgroup, with males possessing a G allele showing, on average, 0.8 more inattentive symptoms than males possessing a C allele (*U* = 1544, *P* = 0.005) (Fig. 2). None of the other SNPs assayed were significantly associated with DSM-IV inattentive, impulsivity or aggressive symptoms (Table 2).

**Table 2:** Relationship between *STS* genotype and DSM-IV symptoms in ADHD males

SNP	Genotype	Inattentive symptoms	Nominal *P* value	Impulsivity symptoms	Nominal *P* value	Aggressive symptoms	Nominal *P* value
rs5934671	C (*n* = 143)	6.98 ± 0.14	0.85	3.26 ± 0.08	0.62	0.48 ± 0.07	0.73
	G (*n* = 74)	6.96 ± 0.21		3.18 ± 0.11		0.42 ± 0.09	
rs2270112	C (*n* = 149)	6.94 ± 0.14	0.62	3.22 ± 0.08	0.79	0.46 ± 0.07	0.82
	G (*n* = 68)	7.05 ± 0.21		3.26 ± 0.11		0.47 ± 0.10	
rs12861247	G (*n* = 186)	6.95 ± 0.13	0.37	3.24 ± 0.07	0.87	0.46 ± 0.06	0.98
	A (*n* = 17)	7.41 ± 0.33		3.24 ± 0.25		0.47 ± 0.21	
rs5978405	T (*n* = 116)	7.04 ± 0.16	0.34	3.33 ± 0.08	0.14	0.52 ± 0.08	0.48
	A (*n* = 85)	6.79 ± 0.20		3.13 ± 0.11		0.40 ± 0.09	
rs17268974	T (*n* = 165)	6.91 ± 0.14	0.51	3.26 ± 0.07	0.46	0.47 ± 0.06	0.87
	A (*n* = 53)	7.17 ± 0.20		3.17 ± 0.13		0.43 ± 0.11	
rs4403552	G (*n* = 141)	7.08 ± 0.14	0.08	3.22 ± 0.08	0.45	0.46 ± 0.07	0.62
	A (*n* = 38)	6.47 ± 0.30		3.08 ± 0.17		0.50 ± 0.13	
rs17268988	C (*n* = 163)	6.90 ± 0.13	**0.03**	3.25 ± 0.07	0.79	0.46 ± 0.06	0.95
	G (*n* = 51)	7.31 ± 0.26		3.24 ± 0.12		0.47 ± 0.12	
rs5933863	G (*n* = 180)	7.10 ± 0.12	0.13	3.23 ± 0.07	0.94	0.50 ± 0.07	0.59
	A (*n* = 36)	6.56 ± 0.32		3.25 ± 0.16		0.33 ± 0.10	

Nominal *P* values <0.05 are highlighted in bold.

### Association between STS variants and cognitive function in male ADHD subjects

Wechsler Intelligence Scales for Children Version III data were not available for eight case individuals due to subject refusal or because they had previously been tested on an alternative IQ test. Of those subjects who were tested, rarely (<5% of the time) data could not be obtained for particular subtests. As with the symptomatology analysis, association with *STS* variants was initially tested in males only (Table 3). One SNP (rs5933863) was highly significantly associated with performance on the picture completion subtest (G: *n* = 171, A: *n* = 36) (*U* = 1909, *P* = 0.0003, *P* < 0.05 after correction for multiple testing), with a second SNP rs4403552 showing suggestive evidence for association with this subtest (G: *n* = 171, A: *n* = 36) (*U* = 3553, nominal *P* value = 0.0021). Significant associations were also noted between rs5978405 and verbal IQ (*t*[185] = 3.556, *P* = 0.000478) and between rs12861247 and comprehension subtest performance (*U* = 1970, *P* = 0.000052), indicated by corrected *P* values <0.05 in each case. These positive results cannot be attributed to age-dependent effects (Table S2).

**Table 3:** Relationship between *STS* genotype and performance on WISC-III measures in ADHD males

SNP	Genotype	PIQ	Nominal *P* value	VIQ	Nominal *P* value	I	Nominal *P* value	C	Nominal *P* value	BD	Nominal *P* value	FFD	Nominal *P* value	PC	Nominal *P* value	CP	Nominal *P* value
rs5934671	C (*n* = 135)	92.0 ± 1.2	0.28	91.4 ± 1.0	0.12	8.8 ± 0.3	**0.035**	8.4 ± 0.3	0.59	8.4 ± 0.3	0.93	89.8 ± 1.2	0.16	10.0 ± 0.2	0.16	8.2 ± 0.3	0.83
	G (*n* = 73)	89.8 ± 1.4		88.6 ± 1.4		8.0 ± 0.3		8.4 ± 0.4		8.4 ± 0.4		87.9 ± 1.4		9.3 ± 0.3		8.2 ± 0.4	
rs2270112	C (*n* = 144)	90.5 ± 1.1	0.30	89.8 ± 1.0	0.30	8.3 ± 0.2	0.083	8.4 ± 0.3	0.85	8.3 ± 0.3	0.57	89.1 ± 1.0	0.71	9.7 ± 0.2	0.66	8.3 ± 0.3	0.41
	G (*n* = 64)	92.6 ± 1.7		91.7 ± 1.6		9.0 ± 0.4		8.5 ± 0.4	8.6 ± 0.4	89.3 ± 1.9		9.8 ± 0.3		7.9 ± 0.4			
rs12861247	G (*n* = 181)	90.9 ± 1.0	0.77	90.3 ± 1.0	**0.033**	8.6 ± 0.2	0.28	8.3 ± 0.2	0.96	8.4 ± 0.2	0.62	89.5 ± 1.0	0.42	9.4 ± 0.2	**0.043**	7.9 ± 0.2	**0.0001**
	A (*n* = 15)	92.3 ± 4.1		97.5 ± 2.6		9.4 ± 0.7		8.1 ± 0.7		8.6 ± 0.9		92.2 ± 3.2		10.9 ± 0.8		10.8 ± 0.5	
rs5978405	T (*n* = 105)	92.5 ± 1.4	0.06	93.5 ± 1.3	**0.000478**	9.1 ± 0.3	**0.0021**	8.7 ± 0.3	0.16	8.4 ± 0.3	0.90	91.9 ± 1.4	**0.016**	9.8 ± 0.3	0.13	8.5 ± 0.3	**0.018**
	A (*n* = 82)	89.0 ± 1.2		87.1 ± 1.2		7.9 ± 0.3		8.0 ± 0.3		8.3 ± 0.3		86.5 ± 1.2		9.1 ± 0.3		7.5 ± 0.3	
rs17268974	T (*n* = 157)	91.0 ± 1.1	0.63	91.1 ± 1.0	0.19	8.8 ± 0.2	**0.024**	8.5 ± 0.3	0.52	8.4 ± 0.2	0.61	89.4 ± 1.1	0.70	9.7 ± 0.2	0.56	8.2 ± 0.2	0.78
	A (*n* = 52)	91.9 ± 1.7		88.5 ± 1.6		7.8 ± 0.3		8.2 ± 0.5		8.5 ± 0.5		88.4 ± 1.5		9.9 ± 0.4		8.1 ± 0.4	
rs4403552	G (*n* = 133)	92.0 ± 1.2	**0.041**	91.8 ± 1.1	**0.020**	8.7 ± 0.2	0.19	8.2 ± 0.3	0.95	8.5 ± 0.3	0.27	90.6 ± 1.1	0.072	10.1 ± 0.2	**0.0021**	8.3 ± 0.3	0.20
	A (*n* = 38)	87.0 ± 1.8		86.5 ± 1.9		8.1 ± 0.4		8.2 ± 0.6		8.1 ± 0.4		86.5 ± 2.2		8.7 ± 0.4		7.6 ± 0.5	
rs17268988	C (*n* = 157)	90.8 ± 1.0	0.36	89.8 ± 1.0	0.15	8.4 ± 0.2	0.11	8.4 ± 0.3	0.68	8.3 ± 0.2	0.27	89.2 ± 1.0	0.35	9.7 ± 0.2	0.49	8.2 ± 0.2	0.87
	G (*n* = 48)	92.8 ± 2.0		92.8 ± 1.9		9.1 ± 0.4		8.3 ± 0.5		8.9 ± 0.5		89.7 ± 2.2		10.0 ± 0.4		8.2 ± 0.4	
rs5933863	G (*n* = 171)	92.2 ± 1.0	0.056	91.5 ± 0.9	**0.024**	8.7 ± 0.2	0.15	8.4 ± 0.3	0.94	8.5 ± 0.2	0.37	89.9 ± 1.0	0.14	10.1 ± 0.2	**0.0003**	8.3 ± 0.2	0.22
	A (*n* = 36)	87.6 ± 1.9		86.4 ± 2.0		7.9 ± 0.5		8.4 ± 0.5		8.2 ± 0.4		87.2 ± 2.2		8.4 ± 0.4		7.7 ± 0.5	

BD, block design; C, coding; CP, comprehension; FFD, freedom from distractibility; I, information; PC, picture completion; PIQ, performance IQ; VIQ, verbal IQ.

Nominal *P* values <0.05 are highlighted in bold.

### Association between STS variants and WAIS-R subtest scores in male healthy volunteer subjects

To simplify data interpretation and to provide maximum comparability with the ADHD data, association between cognitive measures and *STS* variants at SNPs rs5978405, rs5933863 and rs17268988 was initially tested in hemizygous young, male healthy volunteer subjects; cognitive data were obtained for all of these subjects. Data from measures analogous to those in the WISC-III were examined (performance and verbal IQs, verbal information, digit symbol coding, block design, picture completion and verbal comprehension). We detected no evidence for association between the SNPs tested and any of the cognitive measures examined within our healthy volunteer sample (Table 4).

**Table 4:** Relationship between *STS* genotype and performance on WAIS-R subtests in healthy male volunteers ≤30 years of age

SNP	Genotype	PIQ	Nominal *P* value	VIQ	Nominal *P* value	I	Nominal *P* value	C	Nominal *P* value	BD	Nominal *P* value	PC	Nominal *P* value	CP	Nominal *P* value
rs5978405	T (*n* = 84)	118.5 ± 1.4	0.07	119.2 ± 1.5	0.59	18.7 ± 0.4	0.42	63.3 ± 1.0	0.25	41.3 ± 0.6	0.097	14.9 ± 0.2	0.90	22.8 ± 0.3	0.24
	A (*n* = 50)	114.3 ± 1.9		117.9 ± 1.8		18.2 ± 0.5		61.3 ± 1.4		38.9 ± 1.0		14.7 ± 0.3		22.4 ± 0.4	
rs17268988	C (*n* = 91)	116.2 ±1.5	0.88	118.2 ± 1.5	0.84	18.5 ± 0.4	0.23	60.8 ± 1.0	0.089	40.5 ± 0.7	0.19	14.8 ± 0.2	0.90	22.6 ± 0.3	0.41
	G (*n* = 48)	115.8 ± 1.6		117.7 ± 1.8		18.0 ± 0.5		63.8 ± 1.4		39.4 ± 0.8		14.8 ± 0.3		22.4 ± 0.4	
rs5933863	G (*n* = 124)	116.6 ± 1.2	0.56	118.4 ± 1.2	0.52	18.5 ± 0.3	0.62	62.4 ± 0.9	0.13	40.2 ± 0.6	0.48	14.8 ± 0.2	0.76	22.6 ± 0.3	0.28
	A (*n* = 16)	114.6 ± 2.7		116.4 ± 2.8		17.8 ± 1.1		58.5 ± 2.1		39.4 ± 1.5		15.1 ± 0.3		22.2 ± 0.6	

BD, block design; C, coding; CP, comprehension; I, information; PC, picture completion; PIQ, performance IQ; VIQ, verbal IQ.

### In situ hybridization

**Figure 3:**
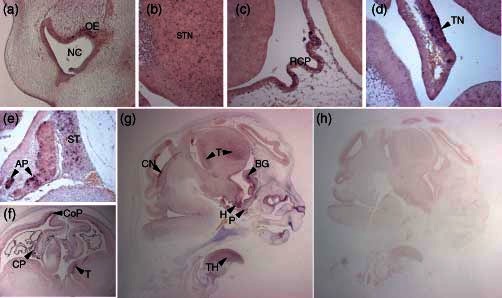
Pattern of *STS* expression (blue staining) in embryonic and fetal human brain (a–e) Sagittal sections from CS18 (44 days of gestation) embryo. (a) OE, olfactory epithelium; NC, nasal cavity (×10 magnification). (b) STN, subthalamic neuroepithelium (×20 magnification). (c) RCP, rhombencephalic choroid plexus (×20 magnification). (d) TN, thalamic neuroepithelium (×20 magnification). (e) AP, anterior lobe of the pituitary gland; ST, sella turcica (×20 magnification). (f) Coronal brain section from CS23 (56–57 days of gestation) embryo. CoP, cortical plate; CP, choroid plexus; T, thalamus (×5 magnification). (g and h) Sagittal sections from subjects at 9 weeks of gestation. (g) T, thalamus; BG, basal ganglia; CN, cerebellar neuroepithelium; H, hypothalamus; P, pituitary gland; TH, thyroid gland (×1 magnification). (h) No blue staining was evident in tissue hybridized with sense riboprobes.

At Carnegie stage 18 (44 days of gestation), *STS* was expressed highly in various epithelial tissues, including cells of the olfactory epithelium, the subthalamic and thalamic neuroepithelia, and in the rhombencephalic choroid plexus (Fig. 3a–d). High levels of expression were also noted in the developing pituitary, particularly the ventral portion of the anterior lobe (Fig. 3e). The gene appeared to be expressed throughout much of the rest of the developing brain, but at much lower levels. Outside the brain, high levels of expression were seen in the developing tongue (Fig. S1). At Carnegie stage 23 (56–57 days of gestation), *STS* was expressed highly in the cortical plate, widely within the developing thalamus and at high levels throughout the choroid plexus (Fig. 3f). At 9 weeks of gestation, high expression levels were observed in the cerebellar neuroepithelium, widely within the thalamus, and also within the basal ganglia, hypothalamus and pituitary gland; lower expression levels were seen in the developing neocortex (Fig. 3g). High expression was also noted in the olfactory epithelium and in the thyroid gland. No hybridization signal was observed in tissue sections hybridized with the sense riboprobe (Fig. 3h).

## Discussion

The primary aim of this study was to examine whether genetic variants within the X-linked *STS* gene, encoding the enzyme steroid sulfatase, were associated with vulnerability to ADHD and with cognitive function in ADHD and healthy subjects. A secondary aim was to map the expression of *STS* during development, with a view to understanding how its altered expression/function might influence ADHD endophenotypes.

Using a case–control approach, we did not replicate the previously reported association between SNP rs12861247 and ADHD, despite having sufficient power to replicate the relatively large reported effect (for SNP association to a complex disorder) (Brookes *et al.* 2008). Both the family study by Brookes *et al.* (2008) and its recent extended follow-up (Brookes *et al.* 2010) were relatively small, and the previously reported association may therefore have been a false positive. However, it is possible that the original finding is a true positive, but that the true effect size is smaller than previously reported, and consequently our analysis was underpowered to detect it. To exclude that possibility very much larger sample sizes are needed. An alternative interpretation is that there are no common variants within *STS* that influence ADHD risk, and that only complete loss of function of the gene (as in the mutant mouse and XLI studies) results in ADHD endophenotypes. It is also important to be aware of two possible limitations of our control sample (from the Wellcome Trust Case Control Consortium): first, it was not specifically matched for IQ to our ADHD sample, although we did exclude ADHD subjects with evidence of learning disability (IQ < 70) from our study. Second, no formal assessment of childhood ADHD symptoms was made in this sample; it is plausible that the presence of subjects with undiagnosed ADHD in the control group may have slightly biased our study toward producing a type II error, although given the incidence of ADHD, this would be expected to have little impact on power (Moskvina *et al.* 2005).

It is also plausible that polymorphisms within *STS* may be associated with a particular ADHD subtype. Our data in mice suggest that a lack of *Sts* results in attentional deficits, but *reduced* impulsivity (Davies *et al.* 2009), while data from XLI subjects suggest that STS dysfunction predisposes mainly to inattentive subtype ADHD (Kent *et al.* 2008). It may therefore be worthwhile testing explicitly for association between *STS* polymorphisms and the inattentive ADHD subtype in a large multicenter sample.

Nevertheless, our data suggest that variation within this gene might influence the phenotypic presentation in ADHD subjects. We replicated the association previously observed between rs17268988 and inattentive symptoms (Brookes *et al.* 2008) in our sample. Specifically, we showed that possession of a G allele at this locus was associated with a greater number of inattentive symptoms, particularly in older boys. This age-dependent effect could be due to the cumulative deleterious effects of possessing this allele or, more speculatively, to some interaction between STS function and steroid hormone biosynthesis around puberty; interestingly, plasma DHEAS concentrations are higher in children older than 9 years of age than in younger children (Smith *et al.* 1989). We have preliminary evidence that variation at rs17268988 is also associated with aspects of cognition (block design) in schizophrenia (data not shown). Variation within *STS* was associated with performance on the picture completion subtest in ADHD males. In this test, subjects must identify a missing item from a series of pieces of artwork within a limited time frame. The task taxes three major psychological processes: distinguishing relevant from irrelevant information, visual alertness and long-term visual memory (Groth-Marnat 1999). Data from mice indicate that visual alertness (‘visuospatial attention’) may be influenced by STS function (Davies *et al.* 2009), while picture completion performance has been suggested as a reasonable predictor of attentional function in children and adults (Fuentes *et al.* 2003; Groth-Marnat & Baker 2003). Moreover, STS inhibition in rats has been shown to elicit effects on spatial memory (Johnson *et al.* 2000). Hence, it is plausible that the observed associations with picture completion performance are consequent upon primary associations with attentional function and/or memory. We also found significant association between *STS* variants and performance on the verbal IQ and comprehension subtests; it is noteworthy that in the latter case, association was seen with rs12861247, the SNP identified as being associated with ADHD and altered frontal cortex gene expression by Brookes *et al.* [specifically, inferior performance was seen in subjects possessing the presumed ‘risk’ allele (G)] (Brookes *et al.* 2008, 2010).

An important potential limitation with studies such as this is the possibility of type I errors. The fact that the results above remain significant upon correction for stringent multiple testing, that the association between rs5933863 and picture completion is replicated in a small sample of ADHD females whereby homozygotes for the G allele significantly outperform heterozygotes and homozygotes for the A allele (data not shown), and that the only subject from previous publications (of whom we are aware) with STS deficiency and ADHD tested using the WISC (family 2, case C) has been reported to show an uneven cognitive profile, with relative strength in picture completion and weakness in comprehension (Kent *et al.* 2008), argues against the reported findings being spurious and suggests that STS may truly influence performance in these areas in ADHD subjects.

We could find no evidence that the three SNPs genotyped in healthy male volunteers were associated with cognitive function. This may be because the associations are only manifest in the context of an abnormally developing brain, because the associations vary with age, because of subtle methodological differences between the WISC-III and the WAIS-R, because any effects within this homogeneous population would be too small to detect with our limited sample size or because the findings from the ADHD sample were indeed false positives. However, as we have argued above, we believe that the latter possibility is unlikely.

*STS* was found to be most highly expressed in various epithelia during development, including the olfactory, thalamic and cerebellar neuroepithelia. High *STS* expression was also observed in the choroid plexus, the hypothalamus and pituitary gland, the thalamus and the basal ganglia. Expression was noted throughout much of the rest of the brain at lower levels, including the developing neocortex. These findings are broadly consistent with previous limited data examining the localization of STS protein/enzyme activity in specific adult brain regions in man (Kriz *et al.* 2008; Steckelbroeck *et al.* 2004), monkeys (Kriz *et al.* 2005) and bovines (Park *et al.* 1997); they are also consistent with data from studies examining the expression of STS in the developing brains of rodents (Compagnone *et al.* 1997) and frogs (Cevasco *et al.* 2009). It is likely that STS activity in the aforementioned brain regions affects their development, and possibly their ongoing function in adulthood. By extension, altered STS expression is likely to adversely affect the development of these structures. Hence, high levels of *STS* expression in the thalamus, basal ganglia and cerebellar neuroepithelium (which gives rise to GABAergic and glutamatergic neurons of the cerebellum; Hoshino *et al.* 2005) were of particular interest, given prior data suggesting aberrant development of these regions in both ADHD (Dickstein *et al.* 2006; Perlov *et al.* 2009; Silk *et al.* 2009; Valera *et al.* 2007) and schizophrenia (Ellison-Wright & Bullmore 2010; Maloku *et al.* 2010). Similarly, altered STS function in the hypothalamus/pituitary gland, olfactory epithelium and tongue could feasibly play a role in the altered hypothalamic-pituitary-adrenal axis responsivity (van West *et al.* 2009), odor detection abnormalities (Karsz *et al.* 2008; Romanos *et al.* 2008) and tongue characteristics (Atmetlla *et al.* 2006) previously described in ADHD.

Recent data from monkeys have shown that the thalamus is a key mediator of visual attention (Rees 2009). Although little is known about the neurobiological correlates of subtest performance on the Wechsler scales, there is some evidence that picture completion performance is relatively specifically affected in subjects with cerebellar lesions (Molinari *et al.* 2004; Ruessmann *et al.* 1989; Schmahmann & Sherman 1998), and is correlated with midline cerebellar volume in adolescents born preterm (Allin *et al.* 2005), implying a role for the cerebellum in performing this cognitive task. Furthermore, picture completion is the most highly correlated WISC-III subtest with basal ganglia volume in children born preterm (Peterson *et al.* 2000), implicating this second brain structure in picture completion performance. In contrast, block design performance does not appear to be markedly affected by cerebellar lesions (Molinari *et al.* 2004), but may be specifically affected by thalamic lesion (Carlesimo *et al.* 2007). Hence, the expression patterns of *STS* are consistent with the associated protein having discrete effects on attention and other aspects of cognition.

Several of the analyses presented herein are exploratory and, as such, should be confirmed in alternative samples. However, when taken together, these data suggest the possibility that variation within *STS* might be associated with altered brain development and affect the severity of inattention symptoms in ADHD. It is possible that there is an association with cognitive function in ADHD, but some caution is required due to testing of multiple phenotypes.

## Supporting Information

**Figure S1:** *STS* expression was detectable (bluestaining) in the mid and surface layers of the tongue at CS18 (44days of gestation) (sagittal section, ×5 magnification).

**Table S1:** The ages of male ADHD subjects with major and minor alleles at rs17268988 at the time of assessment of DSM-IV ADHD symptoms did not differ.

**Table S2:** The ages of male ADHD subjects with major and minor alleles at SNPs rs12861247, rs5978405 and rs5933863 at the time of assessment of WISC-III scores did not differ.

As a service to our authors and readers, this journal provides supporting information supplied by the authors. Such materials are peer-reviewed and may be re-organized for online delivery, but are not copy-edited or typeset. Technical support issues arising from supporting information (other than missing files) should be addressed to the authors.
