# Supplementary material for: Steroid sulfatase is a potential modifier of cognition in attention deficit hyperactivity disorder
Source: Genes Brain Behav. 2011 Apr;10(3):334–44. doi: 10.1111/j.1601-183X.2010.00672.x (PMC3664024; doi:10.1111/j.1601-183X.2010.00672.x)

**Steroid sulfatase is a potential modifier of cognition in Attention Deficit Hyperactivity Disorder**

E. Stergiakouli^1^, K. Langley^1^, H. Williams^1^, J. Walters^1,2^, N.M. Williams^1^, S. Suren^3^, I. Giegling^4^, L.S. Wilkinson^1,2,5^, M.J. Owen^1^, M.C. O’Donovan^1^, D. Rujescu^4^, A. Thapar^1^, W. Davies^1,2,5^*

^1^MRC Centre for Neuropsychiatric Genetics and Genomics and Department of Psychological Medicine and Neurology, School of Medicine, Cardiff University, Cardiff, CF14 4XN, U.K.

^2^School of Psychology, Cardiff University, Cardiff, CF10 3AT, U.K.

^3^Human Developmental Biology Resource, University College London Institute of Child Health, London, WC1N 1EH, U.K.

^4^Section of Molecular and Clinical Neurobiology, Department of Psychiatry, Ludwig Maximilians University, Nussbaumstrasse 7, 80336 Munich, Germany

^5^Behavioural Genetics Group, Schools of Medicine and Psychology, Cardiff University, Cardiff, UK

*to whom correspondence should be addressed:

Henry Wellcome Building,

Heath Park Campus,

Cardiff CF14 4XN

U.K.

Tel: +44-(0)29-2068-7047

Fax: +44-(0)29-2068-7068

e-mail: [daviesw4@cardiff.ac.uk](mailto:daviesw4@cardiff.ac.uk)

**Supplementary Table 1.** The ages of male ADHD subjects with major and minor alleles at rs17268988 at the time of assessment of DSM-IV ADHD symptoms did not differ.

| SNP | Genotype | Age at assessment (months) | Nominal P value |
| --- | --- | --- | --- |
| rs17268988 | C (n = 163) | 111.6 ± 2.1 | 0.96 |
|  | G (n = 51) | 111.1 ± 3.4 |  |

**Supplementary Table 2.** The ages of male ADHD subjects with major and minor alleles at SNPs rs12861247, rs5978405 and rs5933863 at the time of assessment of WISC-III scores did not differ.

| SNP | Genotype | Age at assessment (months) | Nominal P value |
| --- | --- | --- | --- |
| rs12861247 | G (n = 181) | 111.6 ± 1.8 | 0.90 |
|  | A (n = 15) | 111.5 ± 6.2 |  |
| rs5978405 | T (n = 105) | 110.3 ± 2.3 | 0.25 |
|  | A (n = 82) | 113.9 ± 2.5 |  |
| rs5933863 | G (n = 171) | 110.9 ± 1.9 | 0.68 |
|  | A (n = 36) | 113.0 ± 4.5 |  |

**Supplementary Figure 1.** *STS* expression was detectable (blue staining) in the mid and surface layers of the tongue at CS18 (44 days of gestation)(sagittal section, x5 magnification).


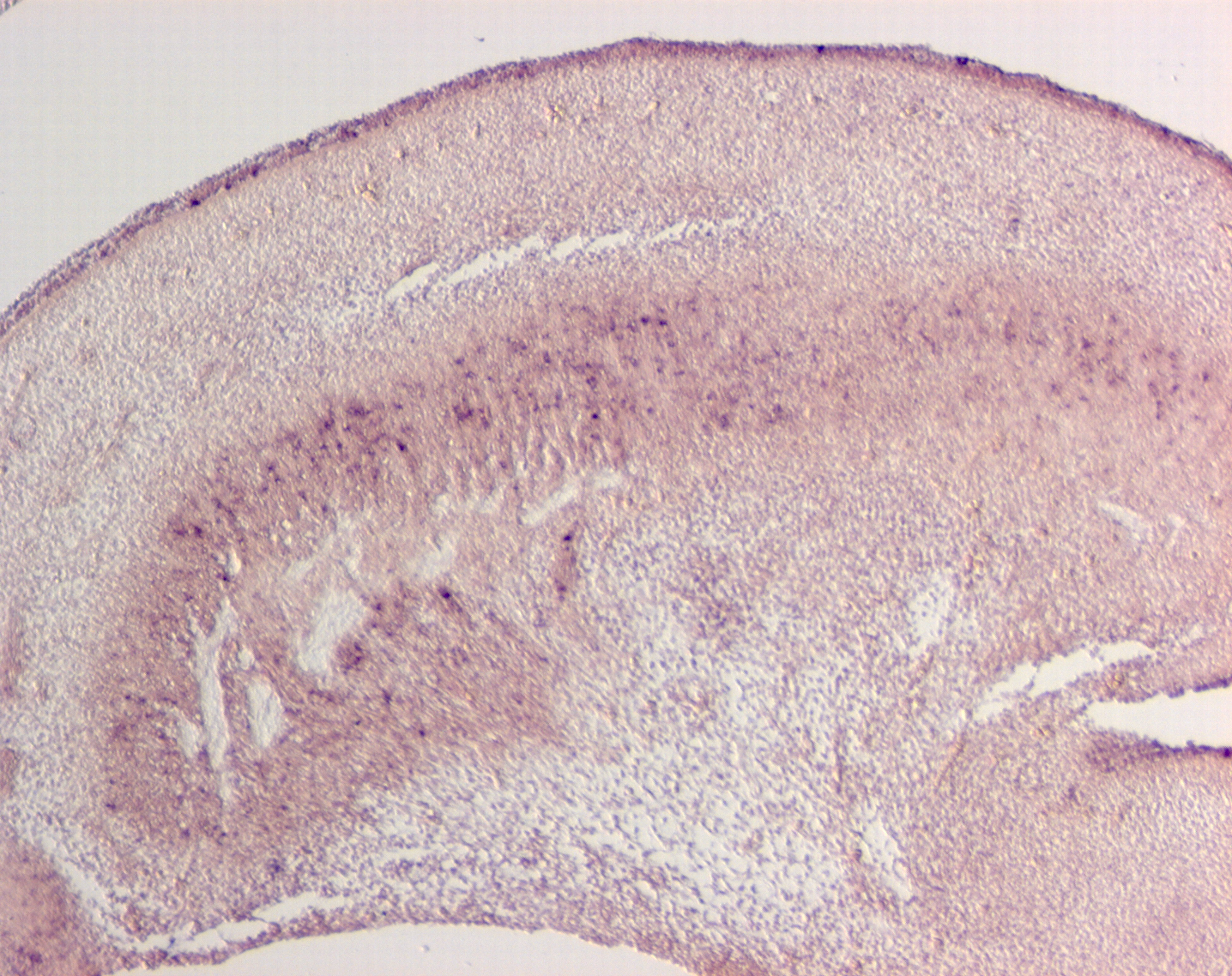

Supplement: Supplementary file 1 [file gbb0010-0334-SD1.doc]
